# The outer membrane in *Acidithiobacillus ferrooxidans* enables high tolerance to rare earth elements

**DOI:** 10.1128/aem.02450-24

**Published:** 2025-04-23

**Authors:** Hannah S. Zurier, Raymond Farinato, Katarzyna H. Kucharzyk, Scott Banta

**Affiliations:** 1 Department of Chemical Engineering, Columbia University 207090 , New York, New York, USA; 2 Department of Earth and Environmental Engineering, Columbia University 539508 , New York, New York, USA; 3 CBRNE Bioscience Center, Battelle Memorial Institute 42786, Columbus, Ohio, USA; University of Nebraska-Lincoln, Lincoln, Nebraska, USA

**Keywords:** *Acidothiobacillus ferrooxidans*, lanthanides, metal toxicity, outer membrane, lipid peroxidation

## Abstract

The development of microbial chassis strains with high rare earth element (REE) tolerance is critical for the advancement of new metal biomining and bioprocessing technologies. In this study, we present a mechanistic understanding of how hyperacidophilic bioleaching organism *Acidithiobacillus ferrooxidans* resists REE-mediated damage at concentrations of REEs as high as 100 mM, while mesophilic *Escherichia coli* BL21 is significantly inhibited by far lower concentrations of REEs (IC_50_ between ~5 µM and ~140 µM depending on the element). Using light microscopy to document physiological changes and fluorescent probes to quantify membrane quality, we prove that cell surface interactions explain REE toxicity and demonstrate its reversibility through the addition of chelators. Removal of the *A. ferrooxidans* outer membrane and cell wall confers REE sensitivity comparable to that of *E. coli*, corroborating the importance of the outer membrane surface. To conclude, we present a model of differential REE sensitivity in the two strains tested, with implications for industrial metal bioprocessing.

**IMPORTANCE:**

Demand for rare earth elements (REEs), a technologically critical group of metals, is rapidly increasing (US Geological Survey, 2024. *Mineral commodity summaries*. Reston, VA). To expand the supply chain without creating environmentally hazardous conditions, there is growing interest in the application of bioprocessing and bioextraction techniques to REE mining and separation. While REE toxicity has been demonstrated in *Escherichia coli* and other mesophilic neutrophiles, the effect of REEs on organisms currently used in metal bioleaching has been less studied. We present physiological evidence suggesting that REEs damage the outer membrane of *E. coli*, resulting in growth inhibition that is reversible by chelation. In contrast, *Acidithiobacillus ferrooxidans* tolerates saturating REE concentrations without apparent inhibition. This study fills gaps in the rapidly expanding body of literature surrounding REE’s impact on microbial physiology. Furthermore, *A. ferrooxidans* resistance to REEs at saturating concentrations (50–100 mM at pH 1.6) is unprecedented in the literature and demonstrates the potential utility of this organism in REE biotechnology.

## INTRODUCTION

The unique physiology of extremophilic organisms enables survival in conditions inhospitable to most forms of life. While most organisms are confined to environments within a limited range of pH values, temperature, osmotic pressure, metal concentrations, and various other parameters, extremophiles not only survive in but require one or more extreme conditions. While extremophilic homologs of industrially important enzymes have been used for decades in process chemistry due to their superior durability (1, 2), it is only through recent genetic engineering advances that extremophilic microbes have emerged as chassis organisms for synthesis of industrially relevant proteins, small molecules, and other useful products (1, 3). Chassis organisms that survive harsh process conditions have a number of applications, including *in situ* processing (1, 4), conversion of low-grade feedstocks to value-added products (5), and bioremediation (6). In addition, because the conditions required by extremophiles are toxic to mesophiles, processes using extremophiles can use fewer contamination controls, reducing the overall cost of the operation (1). Due to the complexity of extremophilic adaptation strategies, it is substantially easier to engineer extremophiles as chassis organisms than it is to engineer more genetically tractable mesophiles to survive extreme conditions (1, 3). The mechanisms by which extremophiles survive their stressful natural environments are therefore of great interest to industrial biotechnology.

While extremophilic adaptations to harsh conditions are tailored to the environment in which they evolved, an emerging body of evidence suggests that there is some inherent flexibility in the stress response that confers cross-tolerance to other extreme conditions (7–10). For example, halophiles resist damage in high salt concentrations due to higher levels of cytosolic antioxidant complexes, which combat desiccation-induced oxidative damage (7). These same antioxidants confer increased durability toward ionizing and ultraviolet radiation levels that would be lethal to most microbes. Organisms that tolerate high concentrations of organic solvents use efflux pumps and other transport mechanisms to rid the cytosol of damaging molecules (8). The same pumps can confer cross-tolerance to antibiotics (8). To maintain a circumneutral internal pH and therefore reduce acid stress, acidophiles have evolved inverted membrane potentials, meaning that it is energetically less favorable for protons to flood the cytosol. Other cations, including heavy metals, are consequentially less toxic to acidophiles (9, 10).

Due to their intrinsic metal tolerance and adaptation to the harsh leaching environment, the most common industrial application of extreme acidophiles is bioleaching (4). *Acidithiobacillus ferrooxidans*, used for redox leaching of sulfide minerals, is the best characterized and one of the most genetically tractable bioleaching organisms (11). Much research has shown that *A. ferrooxidans* is resistant to high concentrations of a variety of transition metals, including iron, copper, cadmium, molybdenum, chromium, and others (12–16). While the primary industrial use of *A. ferrooxidans* is redox-mediated leaching, it has also been shown on a smaller scale to be useful for bioremediation (17–20), electronic waste recycling (21–24), and bioextraction of metals from non-sulfide ores (25, 26). Recently, research has focused on *A. ferrooxidans* leaching and the recovery of rare earth elements (REEs) (27–30).

REEs are a group of technologically critical metals consisting of the lanthanide series, yttrium, and scandium. Demand for REEs has increased substantially over the last decade (31, 32), prompting innovations in extraction and purification technologies (33, 34). The development of biological tools for REE mining and recycling is an area of ongoing research (35–38) and includes microbes such as *A. ferrooxidans*, which leaches REE-bearing ores via acidolysis (29, 30), and other bacteria and fungi that leach via secretion of chelating molecules. REEs have been shown to be toxic to animals (39, 40) and mesophilic microbes (41–44). While it was assumed until recently that REEs had no native biological role (45), certain organisms, primarily methylotrophs, utilize REE cofactors in alcohol dehydrogenase enzymes (45, 46). These organisms have REE storage and trafficking systems that show promise for development in industrial applications (46). Despite this REE dependence, these organisms do not necessarily have high REE tolerance. Indeed, it has been shown that *Pseudomonas putida* KT2440, which utilizes REEs in an alcohol dehydrogenase (47), is as sensitive to REEs as other mesophiles (48). Research on REE tolerance in extremophiles has focused on a limited number of strains and has generally not proposed mechanistic insights (48).

Published mechanistic work on REE toxicity is limited to *Escherichia coli* (44) and indicates that REE-mediated membrane damage leads to cytotoxicity. There is also evidence that free and chelated REEs cross into the cytosol (49–51), though transport and toxicity have not been quantified. There is no published information on how high REE concentrations affect bioleaching microbes, which would be ideal chassis organisms for REE bioprocessing.

In this study, we present a model explaining how *A. ferrooxidans* responds to saturating levels of REEs and reconciles the differential responses that are observed between *A. ferrooxidans* and *E. coli*. Through growth experiments in minimal media, we show that *E. coli* have an REE half-maximal inhibitory concentration (IC_50_) ranging from <10 µM to ~100 µM, depending on the metal, while calcium, which is approximately the same atomic radius as most REEs and binds many proteins interchangeably with REEs, has an IC_50_ of 9.4 mM. In contrast, *A. ferrooxidans* is not observably inhibited by >100 mM REEs. We characterized the response each cell type exhibits to REEs through microscopy and quantitative membrane integrity analysis. The removal of the outer cell membrane significantly increased *A. ferrooxidans* REE sensitivity, leading to the development of a membrane-centered model of *A. ferrooxidans* REE tolerance. The data and model presented have implications for REE bioleaching process development, biotechnologies for metal extraction, and the general understanding of extremophilic stress responses.

## RESULTS

### *E. coli* are highly sensitive to REEs

To establish a baseline understanding of REE toxicity for the experimental setup, we collected growth curves in phosphate-depleted minimal media for laboratory strain and representative mesophile *E. coli* BL21(DE3) for all REEs in concentrations ranging from 1 µM to 1 mM. Since divalent calcium is similar in size and charge density to trivalent REEs, we also collected growth curves with 50 µM to 50 mM calcium under analogous conditions. While calcium is not an ideal control due to its chemical properties (reactivity, solubility, redox lability) differing from REEs, its similar physical properties nonetheless make it useful for comparison. Full growth curves are reported in Fig. S1 (light REEs) and S2 (heavy REEs). IC_50_ values calculated as described in the methods are reported in Table 1, with plots in Fig. S3. Técher et al. observed IC_50_ values in the low micromolar range for *E. coli* grown in REEs in phosphate-depleted minimal media (44), while our data gave IC_50_ values 1–2 orders of magnitude higher. Comparison of the shapes of the growth curves indicates that the *E. coli* used in that study are systemically more sensitive to REEs than the strains used in this study, though the trends are similar. Thus, this discrepancy is likely attributable to the different strains of *E. coli* used as opposed to differences in experimental design or data processing. Overall, we found that REEs inhibit *E. coli* BL21(DE3) growth in phosphate-depleted minimal media with IC_50_ values ranging from <10 µM to ~200 µM.

**TABLE 1:** IC_50_ values for all REEs and calcium on *E. coli* growth

Salt	IC_50_ ± 95% Cl (µM)	Salt	IC_50_ ± 95% Cl (µM)	Salt	IC_50_ ± 95% Cl (µM)
CaCl_3_	5.0 × 10^5^ ± 0.1×10^5^	NdCl_3_	160 ± 170	HoCl_3_	260 ± 390
ScCl_3_	4.3 ± 0.7	Sm_2_(SO_4_)_3_	14 ± 11	ErCl_3_	54 ± 740
YCl_3_	13 ± 10	Eu_2_(SO_4_)_3_	40 ± 32	TmCl_3_	40 ± 24
La_2_(SO_4_)_3_	33 ± 140	GdCl_3_	15 ± 23	YbCl_3_	5.1 ± 1.9
CeCl_3_	75 ± 120	TbCl_3_	93 ± 19	LuCl_3_	140 ± 120
PrCl_3_	200 ± 650	DyCl_3_	17 ± 370		

### *A. ferrooxidans* growth is not inhibited by saturating REEs

**Fig 1:**
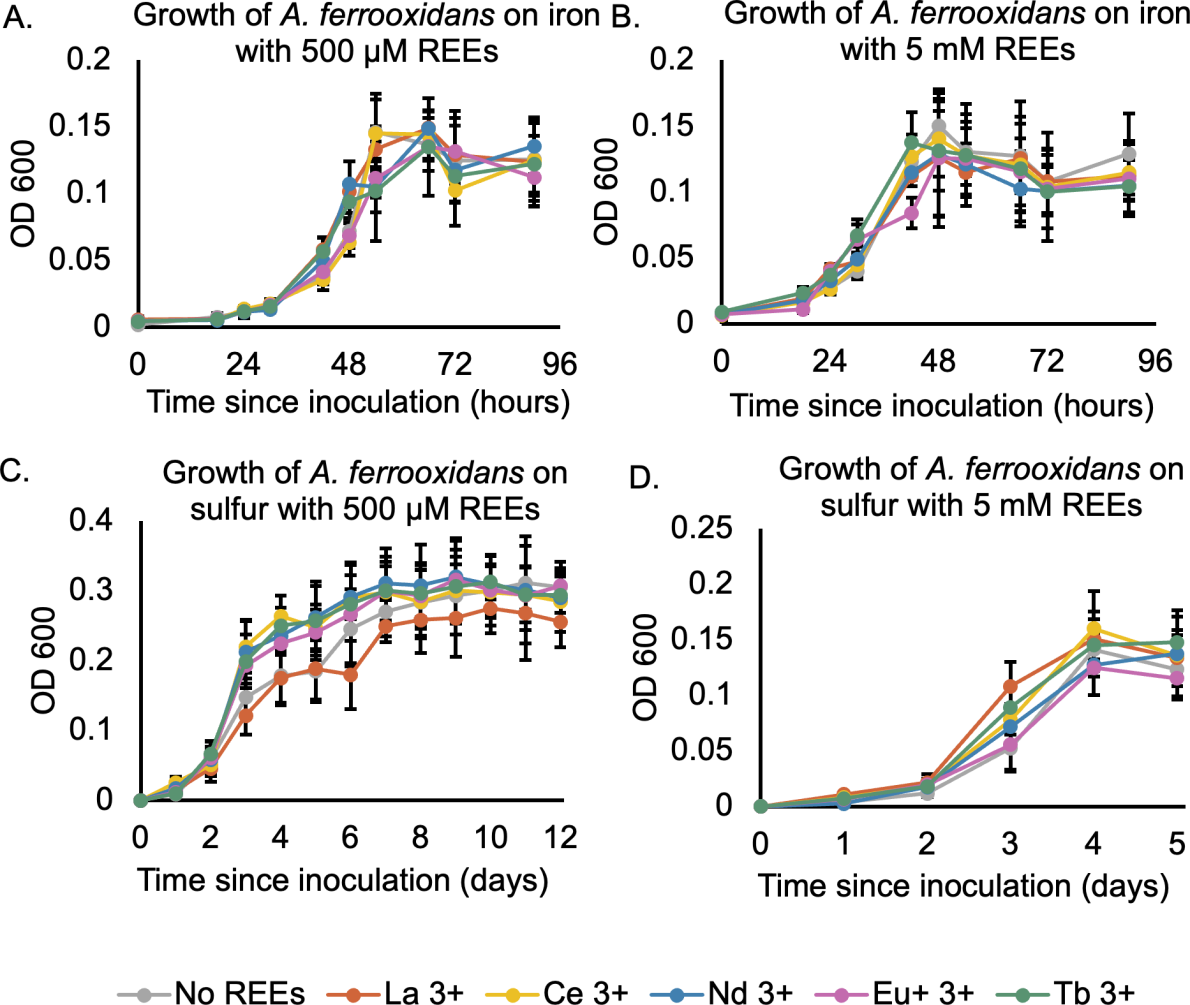
*A. ferrooxidans* is not growth inhibited by REEs on different growth substrates. Cells were grown on either iron (**A and B**) or sulfur (**C and D**), with the indicated amount of rare earth sulfate added to the media. Error bars indicate a standard deviation of three replicates. Time points were taken every 6–24 hours, and cell densities were measured by SYBR green fluorescence, which was then converted to OD 600 as explained in the methods. Since 5 mM is close to the solubility limit for REEs in *A. ferrooxidans* media, we could not determine IC_50_ values in the same manner as we could for *E. coli*. Instead, we attempted to quantify the REE concentration at which *A. ferrooxidans* would stop oxidizing ferrous iron, a proxy for cell integrity and viability. Surprisingly, *A. ferrooxidans* oxidized iron without inhibition in the presence of 100 mM Ho^3+^and Ce^3+^ or 50 mM Nd^3+^ (Fig. 2). Initial growth rates were not affected, with REE-free cultures oxidizing iron at a rate of 12.3 ± 5.5 mM/day, Ce^3+^-containing cultures at 11.1 ± 2.2 mM/day, Nd^3+^ cultures at 13.8 ± 4.3 mM/day and Ho^3+^ cultures at 11.5 ± 1.7 mM/day (Table 2). Without cells present, the ferrous iron oxidized at a significantly lower rate that was also not affected by the presence of REEs (Table 2). This indicates that the cells catalyze the oxidation of ferrous iron to ferric iron and that this process is not affected by the presence of REEs. Because the concentrations studied are close to the saturation point for REE sulfates at pH values consistent with *A. ferrooxidans* iron oxidation, there are likely no conditions in which *A. ferrooxidans* is otherwise viable yet inhibited specifically by REEs.

Our previous work has shown that wild type and engineered *A. ferrooxidans* can accumulate REEs from solutions and may be useful for industrial REE processing (27). Because *A. ferrooxidans* tolerates a variety of heavy metals due to its adaptation to mining environments (12–14, 16), we hypothesized that it may have a high tolerance for REEs as well. We therefore performed growth curves in 500 µM of a selection of individual REEs using either ferrous iron or elemental sulfur as an energy source for the chemolithoautotrophic *A. ferrooxidans* (Fig. 1). While La^3+^ appeared to cause some slight decreases in cell density, none of the REEs had a significant effect on *A. ferrooxidans* growth rates or final cell counts (Fig. 1A and B). This observation was consistent across both media formulations and held as REE concentrations were increased 10-fold to 5 mM (Fig. 1C and D). Indeed, La^3+^ did not show a dose-dependent effect on *A. ferrooxidans* growth, indicating that the apparent inhibition in density at 500 µM was likely due to experimental noise. While 5 mM is approximately 2 orders of magnitude above the *E. coli* IC_50_ for most REEs, *A. ferrooxidans* appeared to be unaffected. While these two strains are metabolically distinct and native to different environments, it nonetheless is useful to compare *A. ferrooxidans* to *E. coli*, which has the best-characterized REE toxicity profile of any microbe and which is a close phylogenetic relative in class *Gammaproteobacteria* (52).

**Fig 2:**
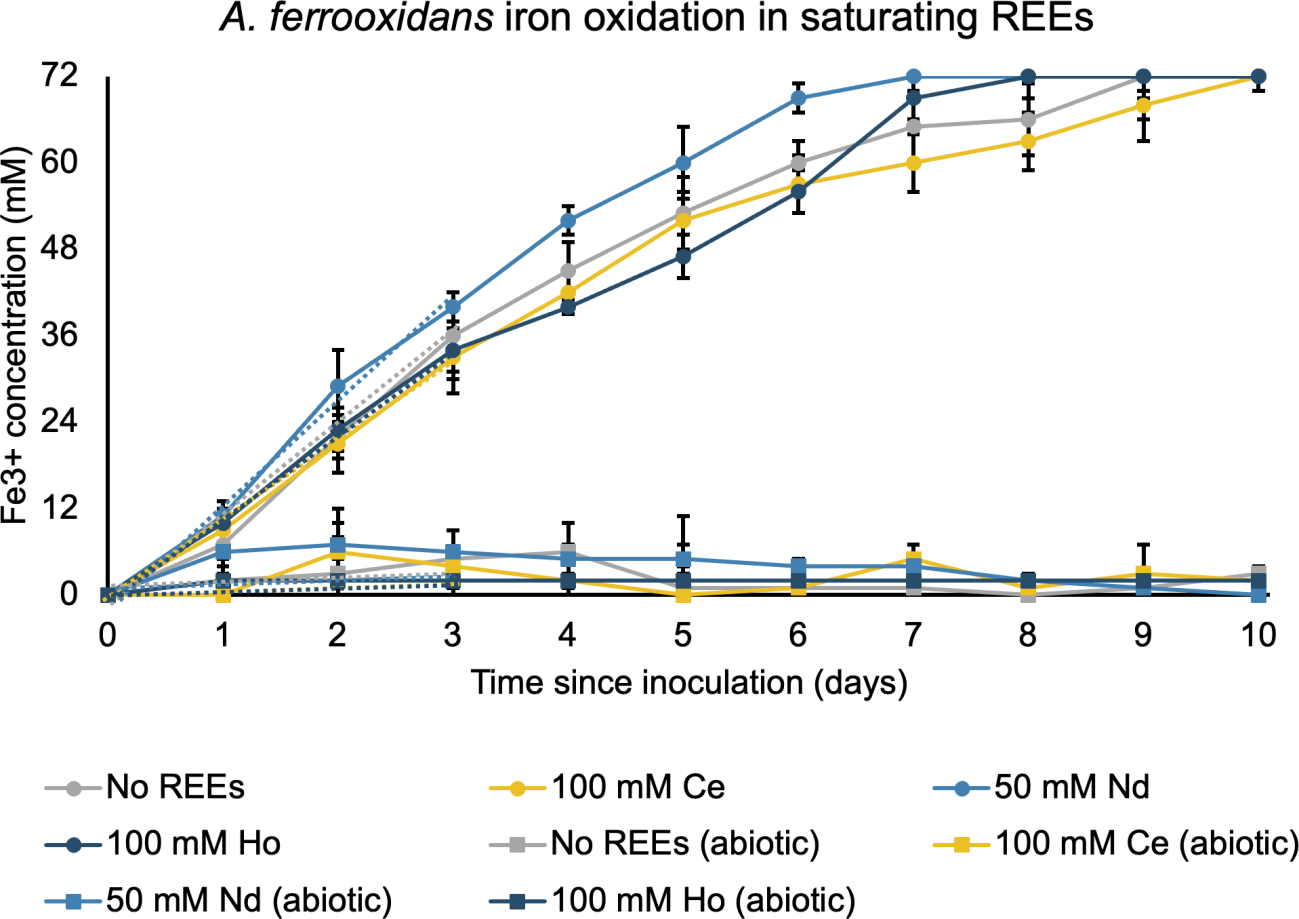
*A. ferrooxidans* is not metabolically inhibited by saturating concentrations of REEs. Ferrous iron at pH 1.6 with the indicated REEs was inoculated with *A. ferrooxidans* to an OD_600_ of 0.025. Iron oxidation was measured every 24 hours. Abiotic samples were not inoculated with *A. ferrooxidans*. Error bars indicate the standard deviation of three replicates.

**TABLE 2:** Initial iron oxidation rates in mM/day ± 95% confidence interval for *A. ferrooxidans* in the presence of saturating REEs

REE	*A. ferrooxidans* Fe^2+^ oxidation rate
100 mM Ce^3+^	11 ± 2
50 mM Nd^3+^	14 ± 4
100 mM Ho^3+^	11 ± 2
No REEs	12 ± 6

### *E. coli* show distinctive, reversible physiological response to REEs

As shown in the growth curves (Fig. S1), when higher REE concentrations (300 µM and 1 mM) are used, data is noisier than when lower concentrations are investigated, despite being read on the same plate reader and grown from the same starter culture in the same base media. This effect may be explained by the fact that in high REE conditions, *E. coli* BL21(DE3) do not grow in a uniform suspension, even with vigorous shaking. Rather, the cells form clumps that settle on the bottom of the wells, creating dense patches surrounded by clear media, which makes OD_600_ a less reliable metric for cell density. We further investigated cell flocculation in the presence of REEs using light microscopy.

**Fig 3:**
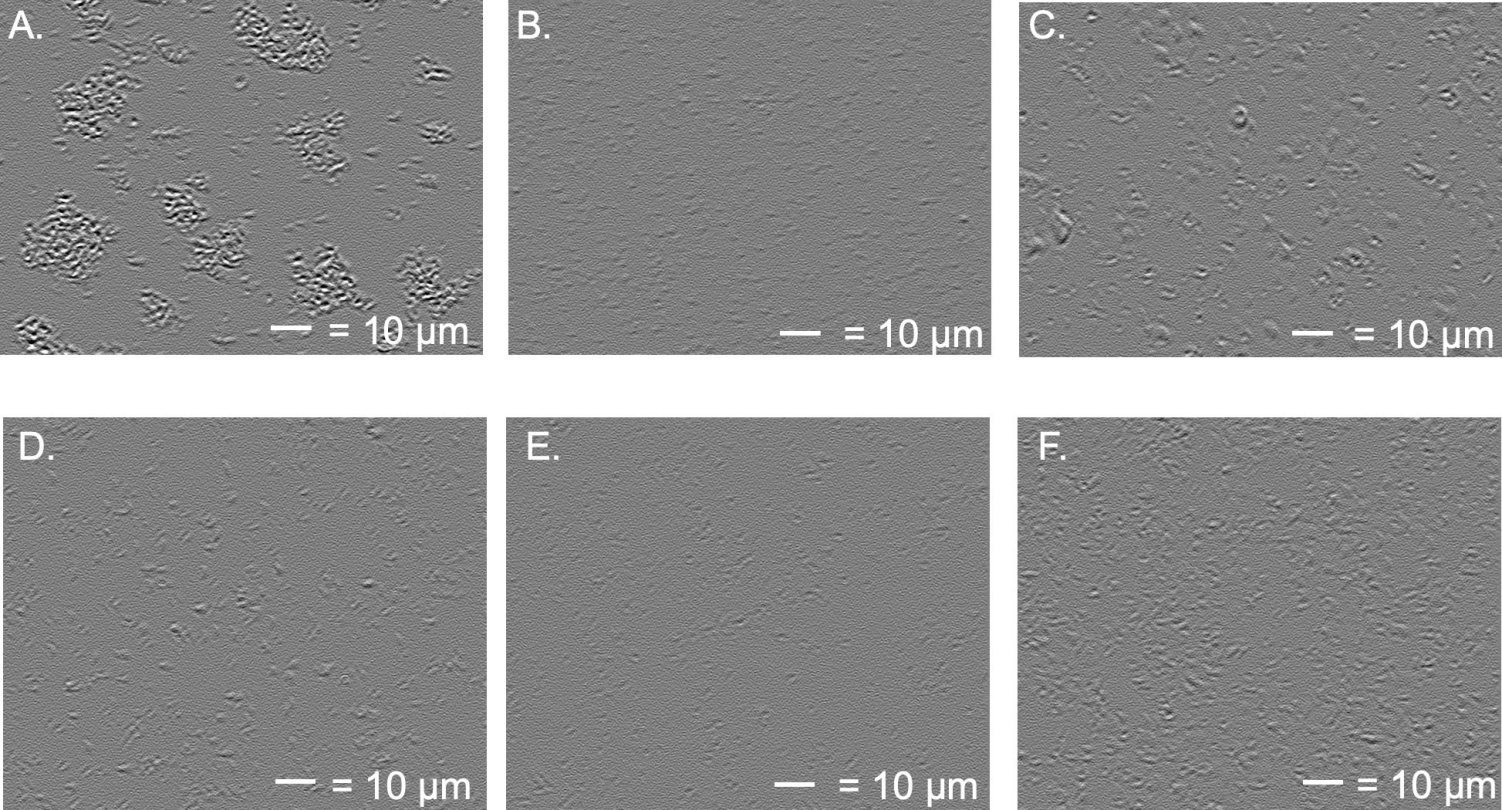
REEs induce EDTA-reversible physiological changes in *E. coli* (panels A, **B, and C**) but not *A. ferrooxidans* (**D, E, and F**). Micrographs are all 1,000×, the scale bar is 10 µm. (A) *E. coli* at physiological pH with 1 mM Tb^3+^ forms clumps containing hundreds of individual cells. (B) *E. coli* at physiological pH with no REEs appears as individual cells in suspension. (C) The addition of EDTA to cell preparation in panel B restores the physiology of panel A. (D) *A. ferrooxidans* appears as individual cells at pH 2 with REEs and without REEs (panel F). (F) *A. ferrooxidans* does not clump in the presence of REEs at pH 7.4.

Under 1,000× magnification, we observed that *E. coli* BL21(DE3) incubated with 1 mM TbCl_3_ for 2 hours formed large (~20 µM) clumps containing ~1,000 individual cells (Fig. 3A). In contrast, cells not exposed to REEs remained distinct and did not form flocs (Fig. 3B). Upon dropwise addition of 500 mM EDTA, clumps in the Tb^3+^-exposed samples immediately broke apart into smaller flocs and individual cells (Fig. 3C). This occurred without mechanical shearing, showing that the presence of EDTA was sufficient to disrupt the flocculation behavior. Because REE phosphates are highly insoluble (53) and previous research has pointed to membrane-REE interactions as being correlated with toxicity (44, 54), we hypothesize that Tb^3+^ ions bind to phosphate groups on the outer *E. coli* membrane, forming patches of positive charge that induce heteroflocculation. EDTA binds terbium more strongly than the phosphates, reversing the flocculation.

**Fig 4:**
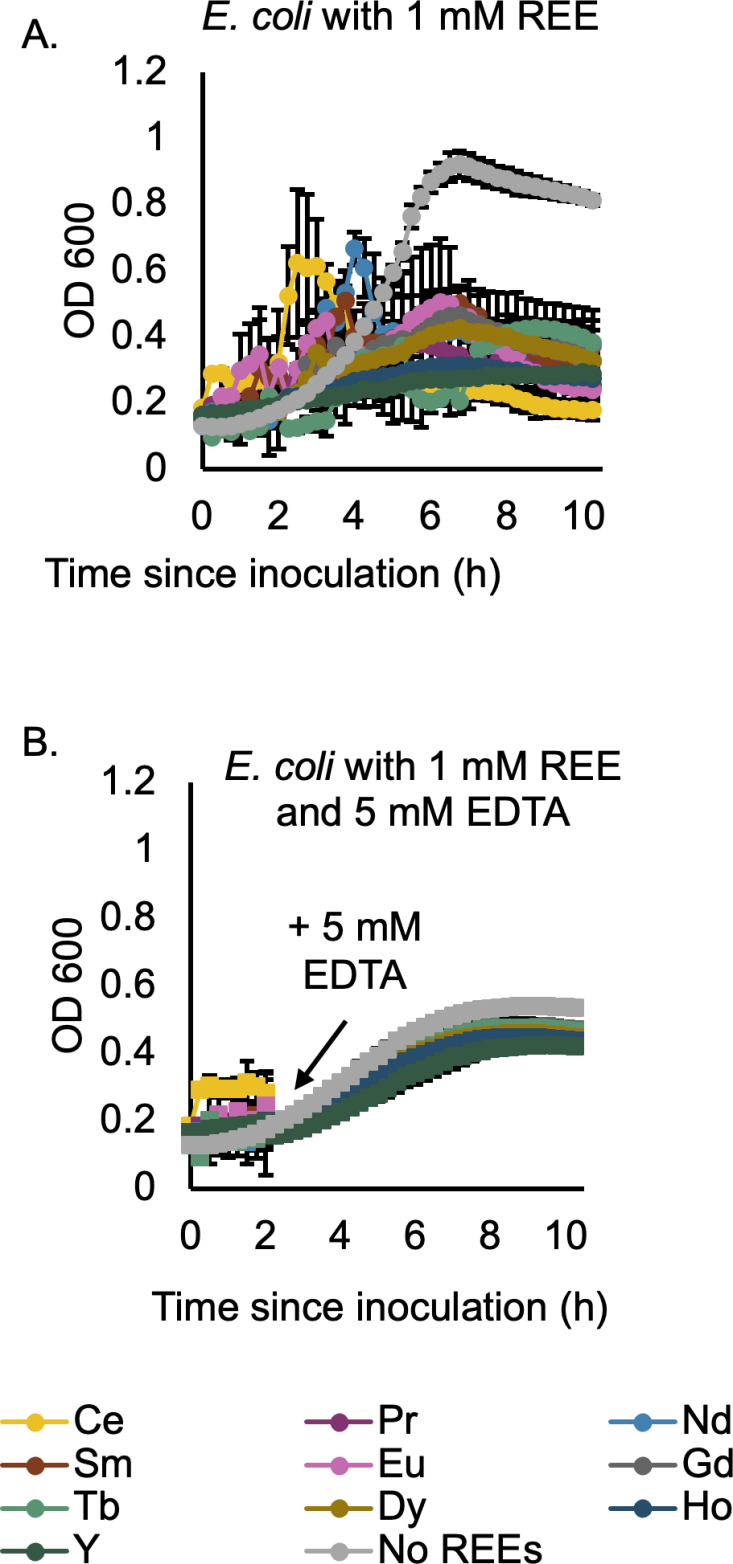
EDTA reverses REE-induced damage in *E. coli*. Growth curves of *E. coli* in phosphate-depleted minimal media with 1 mM of the indicated REE. Error bars indicate the standard deviation of three replicates. (A) REEs inhibit *E. coli* growth. (B) The addition of 5 mM EDTA after 2 hours of growth enables cells to grow normally in the presence of REEs.

To see if reversal of flocculation also reverses REE-mediated growth inhibition, growth curves with and without 1 mM REE were created, and 5 mM EDTA was added to half of the samples after 2 hours of incubation (Fig. 4). The media is engineered to be non-chelating with maximum REE bioavailability (44), so any chelation would come from the added EDTA. While samples without EDTA showed characteristic REE-mediated inhibition (Fig. 4A), EDTA-containing samples showed a reversal of the flocculation behavior (demonstrated by noisy data in growth before EDTA addition), with growth curves mirroring those of cultures not exposed to REEs (Fig. 4B). However, though the curves were of a similar shape and with similar noise to those of cells without REE exposure, the cells grew to a lower density overall (Fig. 4B). Since cells without REE exposure had reduced final density values, it is reasonable to assume that the EDTA is causing some growth inhibition, though not as severely as the REEs. REE-mediated growth inhibition, like REE-mediated flocculation, can therefore be considered reversible upon REE chelation. In addition, since Tb-exposed cells had similar behavior to cells exposed to other REEs, it is reasonable to assume the flocculation effect would also be similar with other REEs.

### *A. ferrooxidans* is not physiologically impacted by REEs

Two potential explanations for the discrepancy in REE sensitivity between *E. coli* and *A. ferrooxidans* are that either (i) REEs cause oxidative membrane damage to *A. ferrooxidans*, as they do to *E. coli*, but the extremophiles have a more robust repair system and therefore do not show noticeable growth defects, or (ii) that REEs do not actually cause damage to *A. ferrooxidans* membranes. To differentiate between these two possibilities, we first used light microscopy to evaluate if *A. ferrooxidans* demonstrated the flocculation behavior of *E. coli* when exposed to REEs. At pH 2, the optimal condition for *A. ferrooxidans*, no REE-induced clumping was observed (Fig. 3E), which could be because REE binding to phosphates is weaker at low pH. We therefore repeated the experiment with *A. ferrooxidans* at pH 7.4, the same conditions we used for *E. coli,* and again did not see flocculation (Fig. 3F). This demonstrates that some fundamental property of *A. ferrooxidans*, likely in the outer membrane, confers REE resistance, supporting hypothesis 2 from above. There is some research showing that *A. ferrooxidans* biofilm surface chemistry is dependent on growth substrate (55, 56), but little is known about the functionality of planktonic cell surfaces.

### REEs quantitatively damage *E. coli* membrane quality

**Fig 5:**
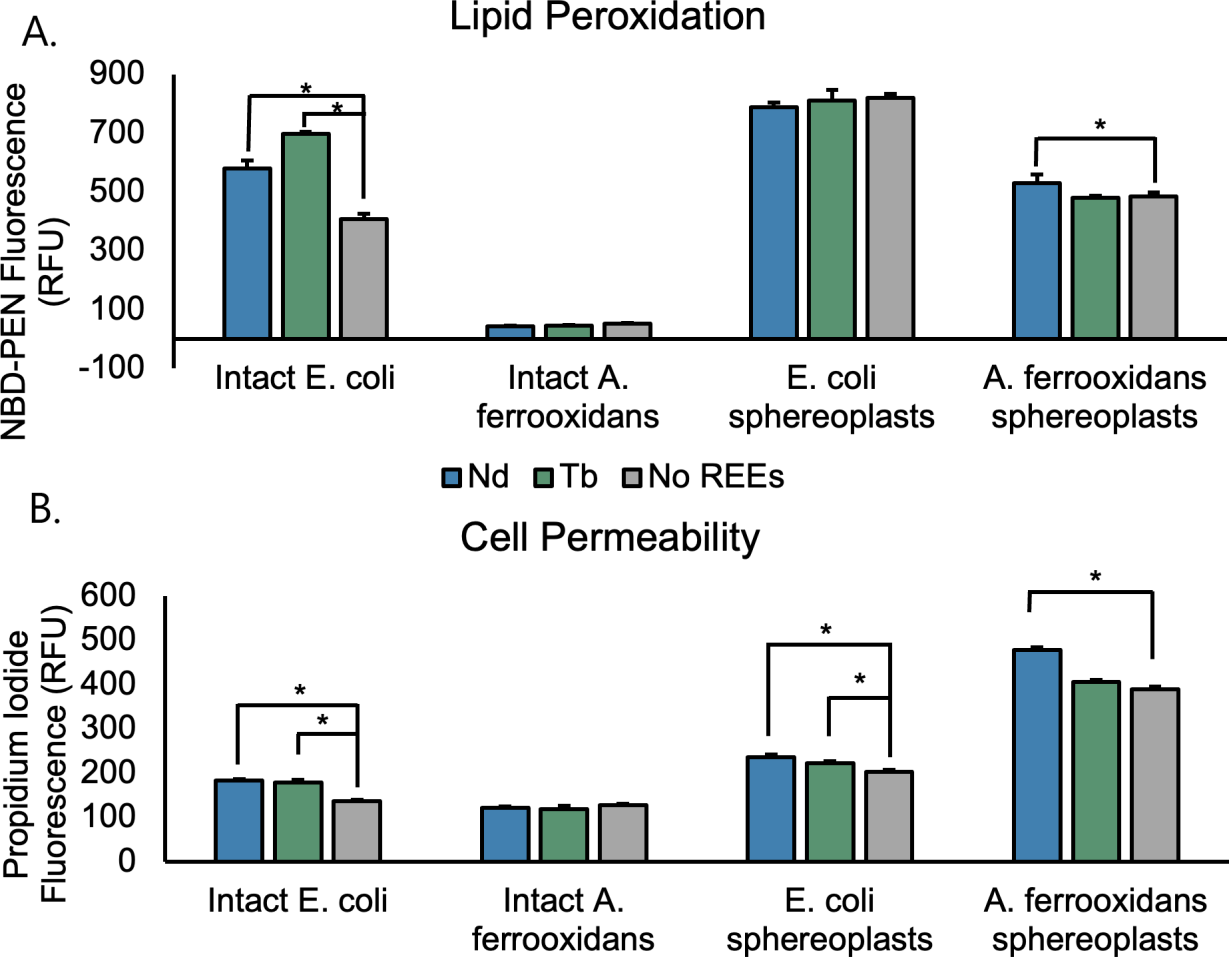
Quantification of REE-induced cell damage. Lipid peroxidation (**A**) was quantified by NBD-PEN fluorescence, and cell permeability (**B**) was quantified by propidium iodide fluorescence. Error bars indicate the standard deviation of three replicates. Asterisks indicate significant differences between samples with and without REEs in the same conditions.

To further investigate the effects of REEs on *E. coli* BL21(DE3)*,* we quantified lipid peroxidation and membrane permeability using fluorescent probes. Exposure to neodymium and terbium both significantly increased oxidative lipid damage (NBD-PEN) (Fig. 5A) and membrane permeability (propidium iodide) (Fig. 5B). Using this information, we propose a model for REE toxicity building on the work of Técher et al. (44), which focuses on membrane damage leading to depolarization and stagnation of ATP synthesis. Our observations of reversible cell flocculation directly causing growth inhibition indicate that lipid peroxidation, in turn leading to membrane damage and cell death, is caused by REE binding to phosphates on the cell surface, creating a high local oxidant concentration that accelerates peroxidation. We further characterized *A. ferrooxidans’* response to REEs through the same fluorescence-based methods. Because all REEs have relatively similar IC_50_ values in *E. coli*, one heavy and one light element were used for this analysis. Neodymium, which has applications in magnets and motors, and terbium, with applications in phosphors and optics, were selected. Lipid peroxidation was not significantly different in cells with and without 1 mM of neodymium or terbium (Fig. 5A). Interestingly, the overall levels of lipid peroxidation were significantly lower in *A. ferrooxidans* than in *E. coli* BL21(DE3), indicating that *A. ferrooxidans* may have a more robust oxidation defense system. A similar trend holds in the propidium iodide assay (Fig. 5B), with *A. ferrooxidans* having overall lower levels of membrane permeability than *E. coli* and with those levels not being affected by 1 mM Nd^3+^ or 1 mM Tb^3+^. Because *A. ferrooxidans* has lower baseline levels of lipid peroxidation and membrane permeability than *E. coli,* these data further support the hypothesis that *A. ferrooxidans* has inherent resistance to damage caused by oxidizing agents like REEs.

### The *A. ferrooxidans* outer membrane is necessary for REE tolerance

Much of the adaptation of *A. ferrooxidans* that has evolved to its extreme natural environment has occurred at its surface. Key genes for the oxidation of sulfur and iron, the cornerstone of *A. ferrooxidans* metabolism, are expressed in the periplasm and span the membranes (57–60). Genes relating to pH tolerance (61), heavy metal resistance (16), and other extreme conditions are similarly localized in the periplasm and membranes. Indeed, *E. coli* and *A. ferrooxidans* are relatively close in evolutionary distance as members of the class *Gammaproteobacteria* despite their differences in metabolism and pH optima (52). Notably, *A. ferrooxidans* membranes contain more cyclopropyl lipids than *E. coli* at a resting state (61, 62), and cyclopropyl lipids are correlated with robust stress response (63). We therefore hypothesized that the outer membrane and periplasm of *A. ferrooxidans* confer much of the observed REE tolerance.

To isolate the role of the outer membrane in *A. ferrooxidans’* resistance to REE-mediated damage, we prepared spheroplasts in both cell lines by digesting the cell wall and dissolving the outer membrane. *E. coli* spheroplasts had the highest magnitude of lipid peroxidation of any samples tested, but lipid peroxidation was not significantly affected by the presence of REEs (Fig. 5A). For cell permeability, *E. coli* BL21(DE3) spheroplasts had similar results to intact *E. coli* in terms of both magnitude and impact of REEs (Fig. 5B). In contrast, this cell preparation rendered *A. ferrooxidans* substantially more sensitive to REEs, with exposure to neodymium significantly increasing lipid oxidation (Fig. 5A) and membrane permeability (Fig. 5B). Terbium did not have any significant effect on either property measured (Fig. 5). Overall, removal of the outer membrane and wall of *A. ferrooxidans* created a REE-induced phenotype similar to that of intact *E. coli* when exposed to REEs, demonstrating that the cell surface of intact *A. ferrooxidans* is crucial to REE tolerance.

## DISCUSSION

**Fig 6:**
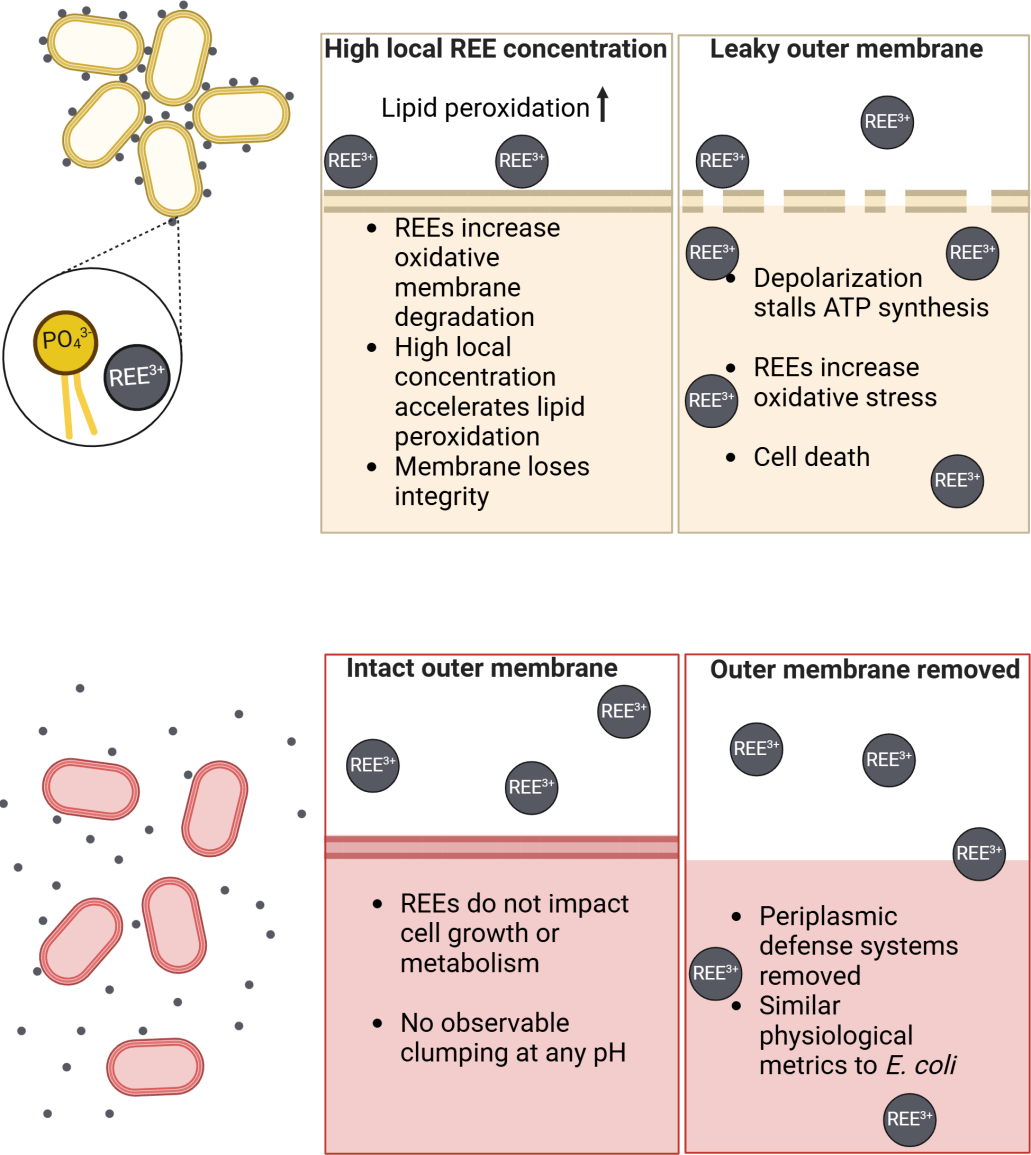
Proposed model of differential REE response in *E. coli* (tan) and *A. ferrooxidans* (red).

To describe the differential response of *E. coli* and *A. ferrooxidans* display toward high REE concentrations, our study proposed the model in Fig. 6. Expanding on the Técher et al. (44) findings that REEs damage *E. coli* membranes, our model includes the evidence presented in this paper that *A. ferrooxidans* resists REE-mediated damage only when the outer membrane is intact. This model is supported by optical microscopy, which shows flocculation with *E. coli* but not *A. ferrooxidans* in the presence of REEs, indicating that the difference between the cell surfaces changes the REE response. Further analysis of REE-exposed *E. coli* and *A. ferrooxidans* using fluorescent probes confirms that only the former shows signs of lipid oxidation and reduced cell integrity. However, removal of the *A. ferrooxidans* outer membrane confers REE sensitivity comparable to that of intact *E. coli* across the three metrics tested. Together, these data suggest that *A. ferrooxidans*, which has a number of membrane-localized adaptations to its extreme natural environment, derives its REE tolerance from structures at the cell surface.

Extremophiles frequently display incidental tolerance toward extreme conditions outside the parameters of their natural habitats. This study demonstrates that *A. ferrooxidans*, naturally acidophilic and tolerant of transition metals, is unaffected in growth and metabolic rate by saturating REE concentrations. REE separation technologies are a rapidly growing field currently dominated by *in vitro* protein-based tools (64, 65). As demand for green processing increases, biological chassis organisms that can thrive under extreme conditions, including low pH and high REE concentrations, will be needed. In addition, organisms with unique genetic modifications may find utility for application-specific bioleaching and biomobilization efforts. Because of its high REE tolerance and genetic tractability, *A. ferrooxidans* is an ideal chassis organism for industrial REE bioprocessing.

## MATERIALS AND METHODS

### Materials

All chemicals were purchased from Sigma-Aldrich (St. Louis, MO, USA) unless otherwise noted. Dispersed sulfur (sold as sulfur, catalog TRC-S789400) was purchased from LGC Standards Ltd. (Teddington, Middlesex, UK). Cerium sulfate octahydrate was purchased from Thermo Fisher Scientific (Waltham, MA, USA). Neodymium sulfate octahydrate and europium sulfate octahydrate were purchased from Fisher Scientific (Hampton, NH, USA). Dysprosium chloride hexahydrate was purchased from Chemscene (Monmouth Junction, NJ, USA). Yttrium chloride was purchased from GFS Chemicals (Powell, OH, USA). NBD-PEN (2,2,6-trimethyl-4-(4-nitrobenzo[1,2,5]oxadiazol-7-ylamino)-6-pentylpiperidine-1-oxyl) was purchased from AOBIOUS, Inc. (Gloucester, MA, USA). Bis-(1,3-dibutylbarbituric acid)trimethine oxonol (DiBAC_4_(3)) was purchased from Thermo Fisher Scientific. BL21(DE3) *E. coli* were purchased from New England Biolabs (NEB, Ipswitch, MA, USA). *Acidithiobacillus ferrooxidans* strain 23270 was purchased from the American Type Culture Collection (ATCC, Manassas, VA, USA).

### Cell culturing

For routine culturing, *E. coli* were grown in an LB medium at 37°C and 150 rpm shaking. To obtain growth curves with REEs, phosphate-depleted minimal media was prepared according to Técher et al*.* (44). The media contained 80 mM NaCl, 20 mM KCl, 20 mM NH_4_Cl, 3 mM Na_2_SO_4_, 0.98 mM MgCl_2_⋅6H_2_O, 0.2 mM CaCl_2_⋅2H_2_O, 20 mM 3-(N-morpholino) propanesulfonic acid, 10 mM sodium 2-glycerophosphate, 28 mM D-glucose and 0.1% (vol/vol) of trace element solution containing 0.51 mM ZnCl_2_ (0.51 mM), 0.62 mM MnCl⋅2H_2_O, 0.97 mM H_3_BO_3_, 0.84 mM CoCl_2_⋅6H_2_O, 0.12 mM CuCl_2_⋅2H_2_O, 0.067 mM NiCl_2_⋅6H_2_O, 0.17 mM Na_2_MoO_4_⋅2H_2_O, 5.5 mM FeCl_3_⋅6H_2_O, in 25% HCl. The media was pH-adjusted to 6.4, filter-sterilized to 0.22 µm, and maintained at room temperature. All *A. ferrooxidans* media contained AF basal salts (66): 0.8 g/L (NH_4_)_2_SO_4_, 2 g/L MgSO_4_⋅7H_2_O, 0.1 g/L K_2_HPO_4_, 0.5% vol/vol Wolfe’s mineral solution (0.5 g/L EDTA, 3 g/L MgSO_4_⋅7H_2_O, 0.5 g/L MnSO_4_⋅H_2_O, 1 g/L NaCl, 0.1 g/L Co(NO_3_)_2_, 0.1 g/L CaCl_2_, 0.1 g/L ZnSO_4_⋅7H_2_O, 0.02 g/L NiCl_2_⋅6H_2_O, 0.01 g/L CuSO_4_⋅5H_2_O, 0.01 g/L AlKSO_4_, 0.01 g/L H_3_BO_3_, 0.01 g/L Na_2_MoO_4_⋅2H_2_O, 0.01 g/L Na_2_WO_4_⋅2H_2_O, 0.001 g/L Na_2_SeO_3_). All media was sterile filtered before use and stored at 4°C. Sulfur was added as appropriate after filtration. For routine culturing, cells were grown in F2S (basal salts supplemented with 10 mM citric acid, 100 mM FeSO_2_, 1 g/L dispersed sulfur, pH 1.8) (66). For sulfur-only media (SM5), basal salt pH was adjusted to 2.0, and 1 g/L dispersed sulfur was added before inoculation. For iron-only media (AFM1), basal salt pH was adjusted to 1.8, and 72 mM FeSO_4_ was added. *A. ferrooxidans* was grown at 30°C and 100 rpm shaking.

### *E. coli* BL21(DE3) growth curves

Stocks of REEs were prepared in ddH_2_O pH-adjusted to 1.8 with concentrated H_2_SO_4_ and maintained at room temperature. Appropriate serial dilutions were made in additional ddH_2_O at pH 1.8. BL21(DE3) *E. coli* were inoculated from glycerol stocks into phosphate-depleted minimal media (see above) and grown overnight at 37°C and 150 rpm shaking. Cells were pelleted by centrifugation (4,695 × *g*, 15 minutes, 4°C) and resuspended in fresh phosphate-depleted minimal media. Optical density was adjusted to 0.1, and 190 µL of cell suspension was added to each well of a clear 96-well plate with lid (Costar, Corning, Inc. Corning, NY, USA). REE solution (10 µL) of an appropriate dilution was added to each well, with negative control wells containing instead 10 µL of ddH_2_O at pH 1.8. The plate was then incubated in a SpectraMax 2 plate reader (Molecular Devices, San Jose, CA, USA) at 37°C with shaking, with OD_600_ recorded at 15 minute intervals for a total of 8 hours. For EDTA-disrupted growth curves, 5 mM EDTA (2 µL of 500 mM stock) was added to the wells after 2 hours of incubation. The plate was then returned to the reader for the remaining 6 hours of the growth curve.

### *A. ferrooxidans* growth curves

REEs stocks were made as described, with only sulfate salts being used due to the sensitivity of *A. ferrooxidans* to halides (67). Stocks of *A. ferrooxidans* were maintained at −80°C in 6% betaine. Starter cultures were prepared in AFM1 media as described above and grown at 30°C with shaking until the media was reddish orange with rust-colored precipitation, indicating complete oxidation of the ferrous iron. All growth curve cultures were inoculated with 1% vol/vol of starter culture in either SM4 or AFM1 media as described above, with REEs added to appropriate concentrations. Cultures were grown at 30°C and 100 rpm shaking. Time points were pulled every 6–12 hours for cells growing on iron and every 24 hours for cells growing on sulfur. To measure cell density, SYBR Green fluorescence was used, as described in Li et al. (68). SYBR Green was purchased as a 10,000× stock in DMSO from Thermo Fisher Scientific and stored at −20°C. A 5× working solution was prepared in tris-EDTA buffer (TE, 1 mM EDTA, 10 mM Tris, pH 8.0) and stored at 4°C protected from light for up to 1 week. For each time point, samples of each culture (1 mL) were removed and spun at 3,000 × *g* for 10 seconds to pellet any solid precipitate. The supernatant containing cells (750 µL) was aspirated and put in a fresh tube. Cells were collected by centrifuging the supernatant at 17,000 × *g* for 1 minute. Spent media was aspirated and discarded. Cells were resuspended in 750 µL TE buffer and incubated at 90°C for 10 minutes to extract DNA. In a black 96-well plate (Costar, Corning), at room temperature for 20 minutes before fluorescence was read (ex. 497 nm, em. 520 nm) in the Spectramax plate reader. To convert fluorescence to OD_600_, background fluorescence (1× SYBR Green in TE buffer) was subtracted from raw values before conversion using the formula OD_600_ = ${\left(\frac{RFU}{72542}\right)}^{\frac{1}{1.6729}}$ (66).

### Measurement of iron oxidation in saturating REEs

Cells were inoculated at 1% vol/vol in 36 mM Fe_2_SO_4_ (72 mM Fe^2+^) and 25 mM Nd_2_(SO_4_)_3_ or 50 mM either Ce_2_(SO_4_)_3_, Pr_2_(SO_4_)_3_, or Ho_2_(SO_4_)_3_ (50 mM or 100 mM REE) at pH 1.6. Incubation was done at 30°C and 150 RPM. Conversion of Fe^2+^ to Fe^3+^ was measured by cerimetry titration as described (68). Samples of cultures (1 mL) were taken every 24 hours and centrifuged at 17,000 × *g* for 1 minute to separate cells, and the supernatant was used for further analysis. Ferroin indicator (10 µL) was added to each sample, and 100 mM Ce^4+^ was added in 10 µL increments until the indicator changed from red to colorless, indicating the complete oxidation of Fe^2+^. Each 10 µL of Ce^4+^ corresponded to 1 mM Fe^2+^ in solution, and from this, concentrations of Fe^2+^ and Fe^3+^ were calculated.

### Microscopy

*E. coli* was grown in LB media overnight, pelleted by centrifugation, and resuspended in buffer (150 mM NaCl, 50 mM Tris, pH 7.4) to an OD of 1. *A. ferrooxidans* was grown in F2S media until iron was completely oxidized, approximately 3–5 days, and pelleted by centrifugation. Cells were separated from residual sulfur through initial centrifugation at 3,000 × *g* for 10 seconds. The sulfur pellet was discarded, and the cells in the supernatant were pelleted at 17,000 × *g* for 1 minute. The pellet was washed three times in basal salts as described above containing 10 mM citric acid at pH 1.8, at which point all residual ferric iron was chelated, and the supernatant was clear. At this stage, the cells were resuspended in basal salts without citrate at pH 2.0 to an OD_600_ of 1. For REE-containing samples, TbCl_3_ was added to a final concentration of 1 mM. All samples were incubated at 30°C and 100 RPM shaking for 2 hours. At this point, *E. coli* were visibly sinking to the bottom of the tubes, so cultures were resuspended by aspiration before being applied dropwise to glass microscope slides and covered with coverslips. Slides were analyzed immediately after preparation on a VHX-970FN digital microscope (KEYENCE, Itasca, Il, USA) under 1,000× magnification. To observe the effect of chelators, separate drops of cell suspension and 500 mM EDTA were placed on slides and covered by a coverslip to create three distinct zones: cells without EDTA, EDTA without cells, and a diffusion zone in the middle where cells could interact with the EDTA. Images and videos were captured using the proprietary VHX software. The images shown were digitally processed using VHX software to enhance edge contrast.

### Fluorescent assays with intact cells

Stocks of NBD-PEN (5 mM), DiBAC_4_(3) (30 mM), and propidium iodide (3 mM) were prepared in DMSO and stored protected from light at −20°C when not in use. Working solutions at 5× target concentration (5 µM NBD-PEN, 125 µM DiBAC_4_(3), 150 µM propidium iodide) were prepared in ddH_2_O immediately before use. *E. coli* were grown for ~18 hours in LB broth and pelleted at 4,695 × *g* for 15 minutes. Pellets were washed in 2:2 basal salts (22.5 g/L (NH_4_)_2_SO_4_, 3.75 g/L MgSO_4_·7H_2_O, 0.75 g KCl, sterile filtered and stored at room temperature) and resuspended to an OD_600_ of 5. *A. ferrooxidans* were grown in F2S medium for 3–4 days until all iron was oxidized (determined by media color shift from yellow to dark brown) and pelleted at 4,695 × *g* for 7 minutes. Cells were resuspended in AF basal salts with 10 mM citric acid at pH 1.8, and the suspension was centrifuged at 3,000 × *g* for 10 seconds to pellet residual sulfur. The supernatant containing cells was transferred to a separate tube and spun at 17,000 × *g* for 1 minute to pellet cells. The pellet was washed 3× in the citric acid basal salts and then 3× in 2:2 basal salts pelleting at 17,000 × *g* for 1 minute and discarding any residual sulfur at each resuspension. Cells were resuspended in the third 2:2 basal salts wash and adjusted to an OD_600_ of 5. REEs (1 mM either TbCl_3_ or NdCl_3_) were added to the cell suspension, and cells were incubated at 30°C for 2 hours with 100 rpm shaking. After incubation, cells were pelleted at 17,000 × *g* for 1 minute, washed 1×, and then resuspended in fresh 2:2 basal salts without REEs. For propidium iodide, 200 µL cell suspension was combined with 50 µL propidium iodide working solution in the wells of a black 96-well plate and incubated statically under aluminum foil for 30 minutes before reading fluorescence at 535 nm excitation and 617 nm emission in the SpectraMax 2 plate reader. For NBD-PEN and DiBAC_4_(3), cells were diluted 10-fold in 2:2 basal salts before 200 µL suspension was combined with 50 µL working solution in each well of a black 96-well plate and incubated at room temperature under aluminum foil for 10 minutes before fluorescence was read (ex. 470 nm/em. 530 nm for NBD-PEN; ex. 490 nm/em. 516 nm for DiBAC_4_(3)).

### Fluorescent assays with spheroplasts

Spheroplasts were prepared as described (69). Cells were harvested and washed as above, with the final resuspension in 3 mL 20% sucrose. To prepare spheroplasts, the following were added in order: 1.5 mL 2 M sucrose, 1.7 mL 0.1 M Tris-HCl (pH 8.0), 0.13 mL 1% EDTA, 0.15 mL 10 mg/mL lysozyme. Samples were subsequently incubated with 100 rpm shaking at 30°C for 1 hour. To harvest spheroplasts, samples were centrifuged at 15,000 × *g* for 15 minutes. Spheroplast pellets were resuspended in 2:2 basal salts supplemented with 0.8 M sucrose, and REEs were added, followed by incubation as described above. After washing in 2:2 basal salts, cells were resuspended in 2:2 basal salts with 0.8 M sucrose before the addition of fluorescent probes and quantification as above.

### Statistical analysis

Quantitative measurements were all performed in triplicate and reported as mean ± standard deviation. To calculate IC_50_ values, growth curves were fitted to a Gompertz model using MATLAB 2024a (MathWorks Inc., Natick, MA, USA), which gave four parameters with associated confidence intervals. The parameter d, corresponding to the lower asymptote of the function and the initial inoculum of the culture, was subtracted from parameter a, which models the upper asymptote of the function and maximum OD of the culture, to generate parameter ad, corresponding to the total change in OD over the course of the growth period. Error was propagated using standard methods. Parameter ad and associated error values for each concentration tested were fitted to a 4-parameter logistic model using MATLAB 2024a to generate IC_50_ values. Values corresponding to growth curves with a poor fit to the Gompertz model were not used in the calculation of IC_50_ values. Plots showing the fitting of the 4-parameter logistic model for each REE are included in Fig. S3. IC_50_ is reported as the fitted value ±95% confidence interval in Table 1. Other *P*-values were determined using a Student’s two-tailed t test with α = 0.05.

## SUPPLEMENTAL MATERIAL

Supplemental figures aem.02450-24-s0001.pdf Figures S1 to S3.

ASM does not own the copyrights to Supplemental Material that may be linked to, or accessed through, an article. The authors have granted ASM a non-exclusive, world-wide license to publish the Supplemental Material files. Please contact the corresponding author directly for reuse.
