# Supplementary material for: The outer membrane in Acidithiobacillus ferrooxidans enables high tolerance to rare earth elements
Source: Appl Environ Microbiol. 2025 Apr 23;91(5):e02450-24. doi: 10.1128/aem.02450-24 (PMC12093958; doi:10.1128/aem.02450-24)
Supplement: Supplemental figures — Figures S1 to S3. [file aem.02450-24-s0001.pdf]

Supplemental Materials:

The outer membrane in *Acidithiobacillus ferrooxidans* enables high tolerance to rare earth elements

Hannah S. Zurier[a], Raymond Farinato[b], Katarzyna H. Kucharzyk[c,] and Scott Banta[a,]*

[a] Department of Chemical Engineering, Columbia University, New York, NY

[b] Department of Earth and Environmental Engineering, Columbia University, New York, NY

[c] CBRNE Bioscience Center, Battelle Memorial Institute, Columbus, OH

*Corresponding author. Mailing address: Department of Chemical Engineering, Columbia University, 820 Mudd MC4721, 500 W. 120th St., New York, NY 10027. Phone: (212) 854-7531, Fax: (212) 854-3054. E-mail: sbanta@columbia.edu

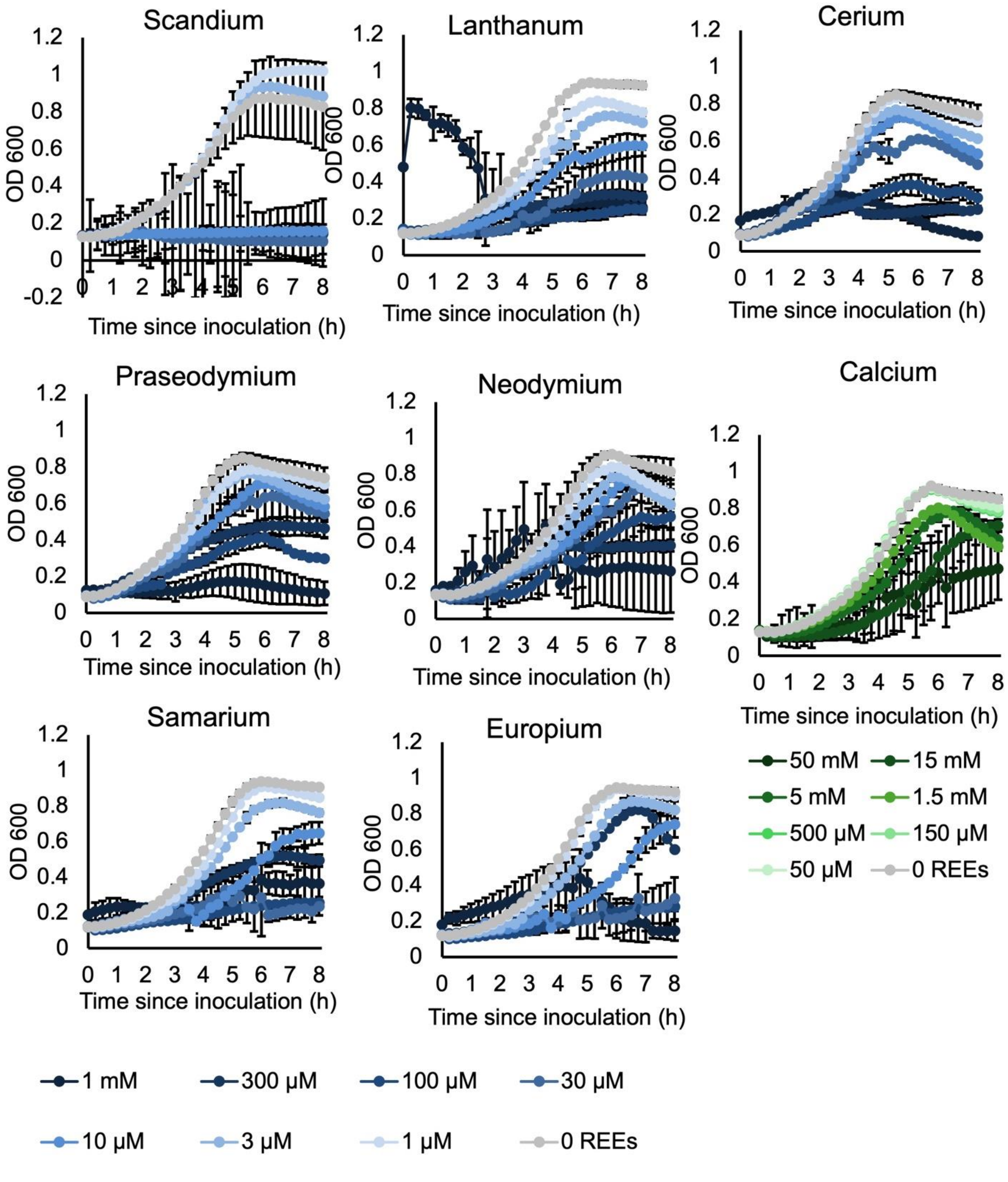

Supplementary Figure 1: Growth curves of *E. coli* with light REEs and calcium
Cells were grown in phosphate-depleted minimal media supplemented with the indicated metal at the indicated concentrations. Initial OD_{600} was 0.15. Error bars indicate standard deviation of 3 replicates. Timepoints were taken every 15 minutes for 8 hours.

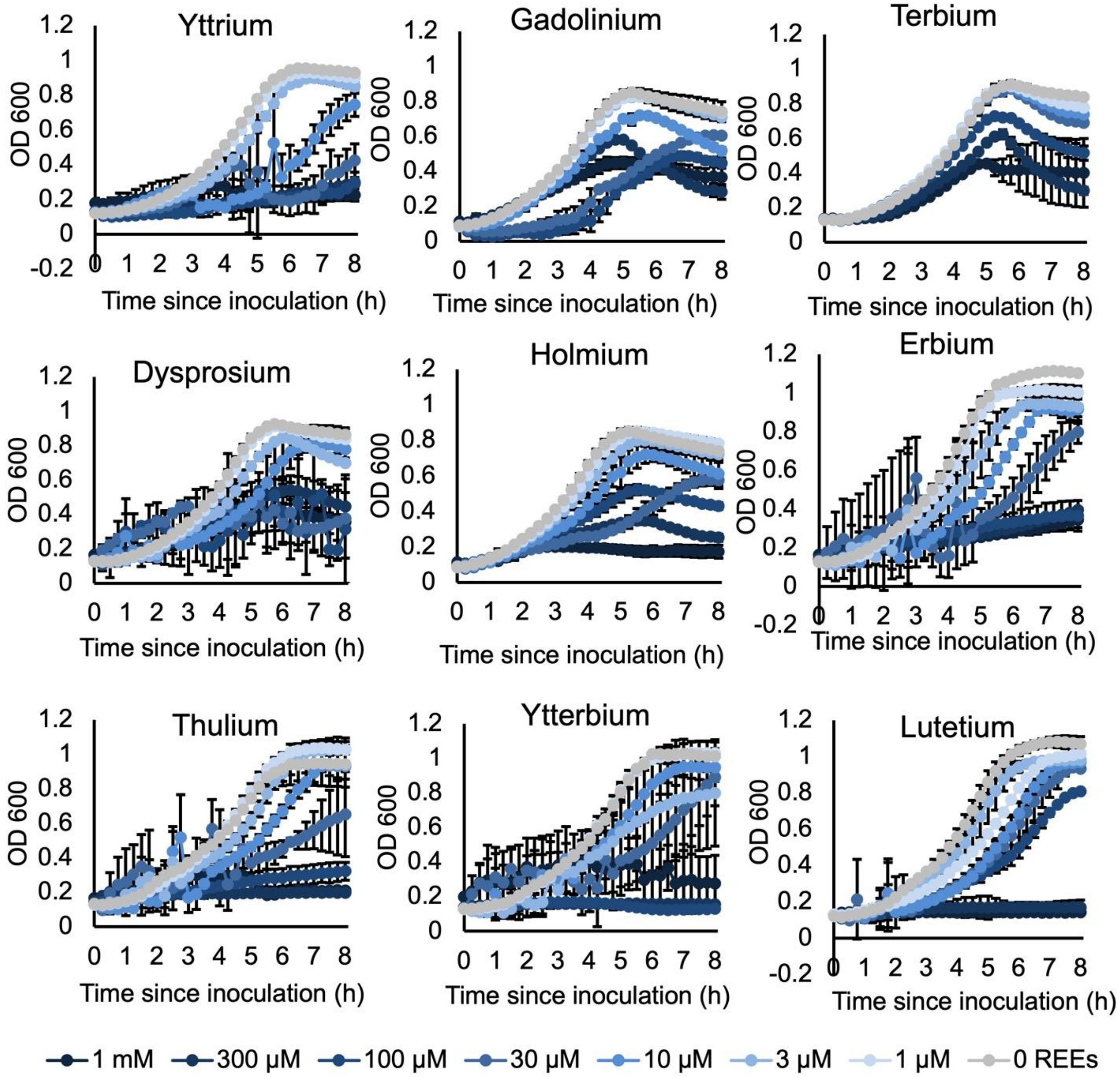

Supplementary Figure 2: Growth curves of *E. coli* with heavy REEs

Cells were grown in phosphate-depleted minimal media supplemented with the indicated metal at the indicated concentrations. Initial OD_{600} was 0.15. Error bars indicate standard deviation of 3 replicates. Timepoints were taken every 15 minutes for 8 hours.

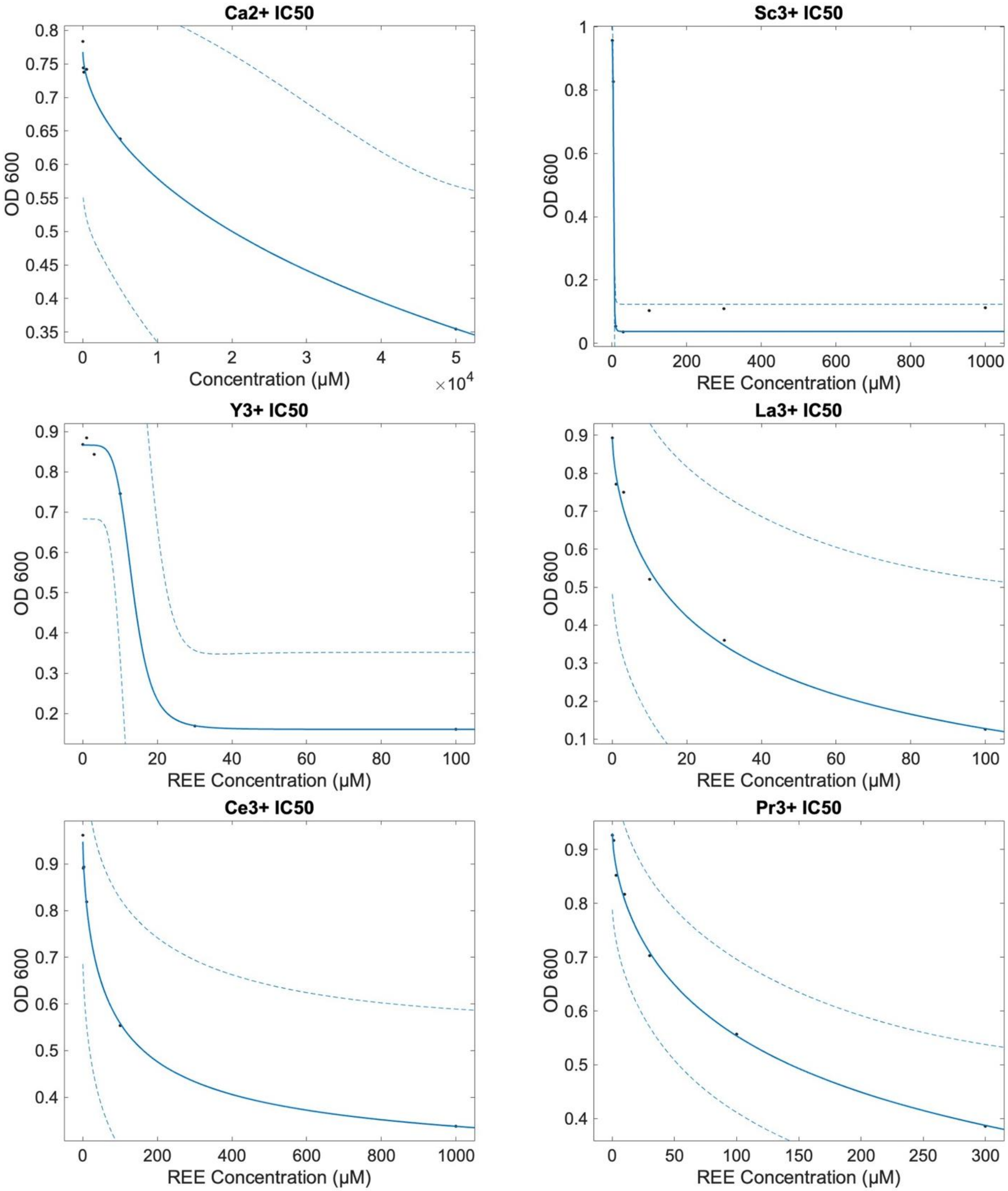

Ca2+ IC50
OD 600
Concentration (μM)
×10^4
Sc3+ IC50
OD 600
REE Concentration (μM)
Y3+ IC50
OD 600
REE Concentration (μM)
La3+ IC50
OD 600
REE Concentration (μM)
Ce3+ IC50
OD 600
REE Concentration (μM)
Pr3+ IC50
OD 600
REE Concentration (μM)

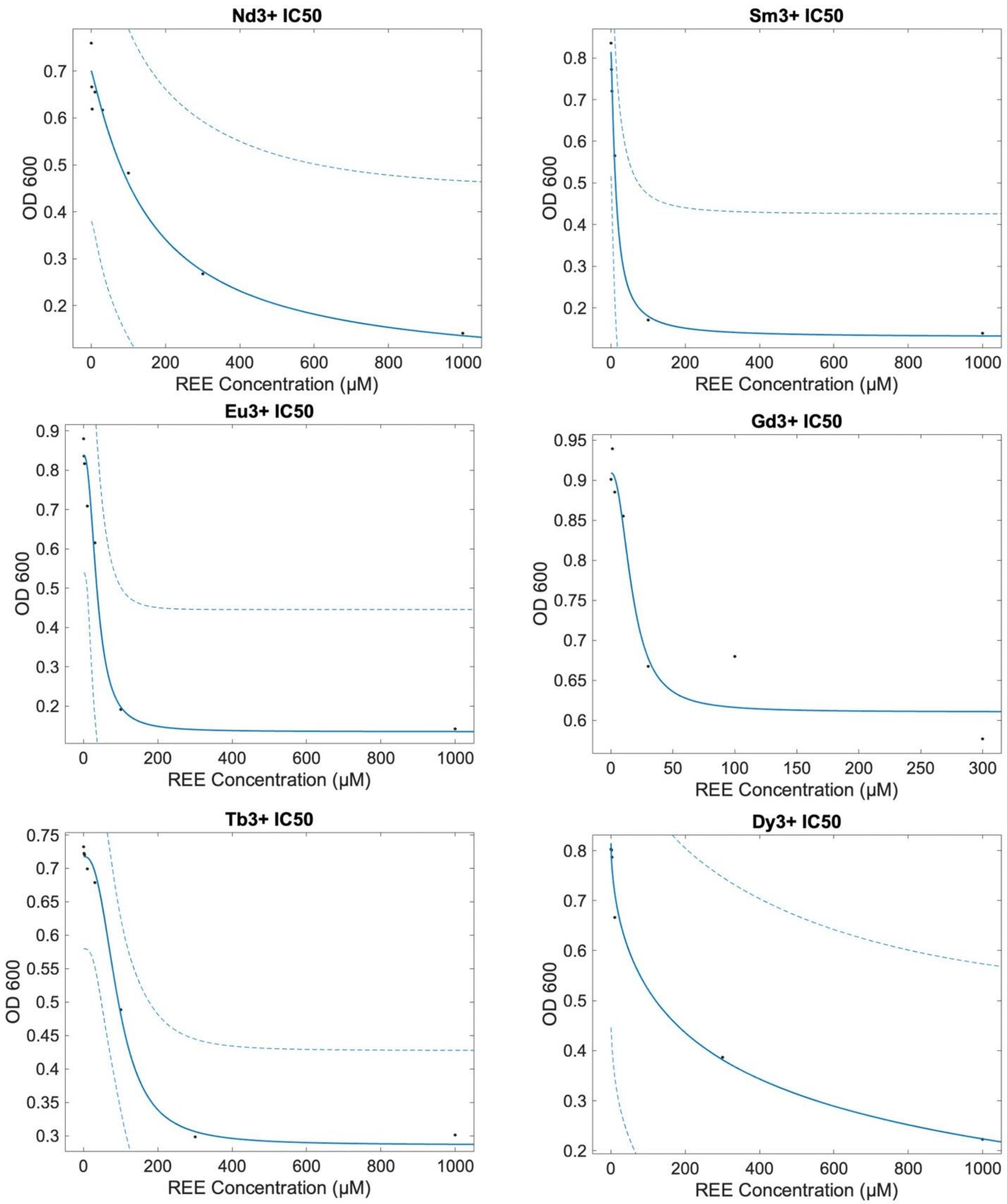

Nd3+ IC50
OD 600
0.7
0.6
0.5
0.4
0.3
0.2
0
200
400
600
800
1000
REE Concentration (μM)
Sm3+ IC50
OD 600
0.8
0.7
0.6
0.5
0.4
0.3
0.2
0
200
400
600
800
1000
REE Concentration (μM)
Eu3+ IC50
OD 600
0.9
0.8
0.7
0.6
0.5
0.4
0.3
0.2
0
200
400
600
800
1000
REE Concentration (μM)
Gd3+ IC50
OD 600
0.95
0.9
0.85
0.8
0.75
0.7
0.65
0.6
0
50
100
150
200
250
300
REE Concentration (μM)
Tb3+ IC50
OD 600
0.75
0.7
0.65
0.6
0.55
0.5
0.45
0.4
0.35
0.3
0
200
400
600
800
1000
REE Concentration (μM)
Dy3+ IC50
OD 600
0.8
0.7
0.6
0.5
0.4
0.3
0.2
0
200
400
600
800
1000
REE Concentration (μM)

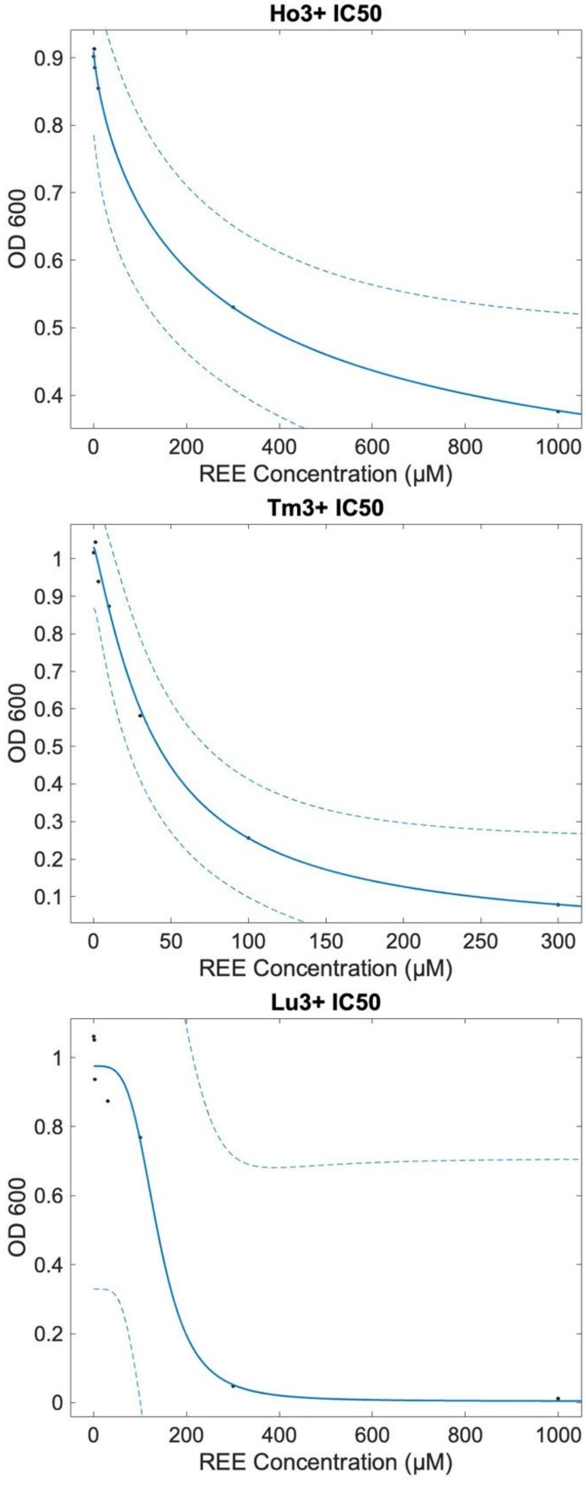

Supplementary Figure 3: Fitting growth curve parameters to a logistic model. For all growth curves in figures S1 and S2, parameter ad was derived from a Gompertz fit of OD vs time (parameter d, corresponding to the bottom asymptote subtracted from parameter a, corresponding to the top asymptote; method described in detail in methods section). Parameter ad was then fit as shown to a four-parameter logistic model to derive IC_{50} values shown in table 1.
